# 1570. Exploring Antiretroviral Therapy (ART) Switch Decisions in Clinical Setting: Trio Health Mixed Methods Study

**DOI:** 10.1093/ofid/ofad500.1405

**Published:** 2023-11-27

**Authors:** Rick A Elion, Megan Dunbar, K Rivet Amico, Joshua Gruber, Lauren Harrison, Grace A McComsey, Peter Shalit, Janna Radtchenko

**Affiliations:** Trio Health, Louisville, Colorado; Gilead Sciences, Forest City, California; University of Michigan School of Public Health, Ann Arbor, Michigan; Gilead Sciences, Forest City, California; University of Michigan School of Public Health, Ann Arbor, Michigan; Case Western Reserve University , Cleveland, OH; Trio Health, Louisville, Colorado

## Abstract

**Background:**

This study describes clinical decisions on switching to B/F/TAF or DTG/3TC.

**Methods:**

Sequential explanatory design was used in this mixed methods study. Retrospective EMR analysis of Trio HIV Network data characterized people living with HIV (PWH) ≥18 yrs prescribed B/F/TAF or DTG/3TC (4/2019-6/2022). Informed by retrospective analyses, interviews among selected clinicians were summarized through thematic analyses. Qualitative responses were then quantified to describe regimen choice.

**Results:**

In EMR analysis, of 6996 eligible PWH, 84% were prescribed B/F/TAF, 16% DTG/3TC. B/F/TAF prescription was associated with HIV clinical parameters (detectable viral load [VL], CD4< 200 cells/μL) and substance use, while prescribing DTG/3TC was associated with renal toxicity and obesity.

In qualitative analysis, 27 clinicians (44% MDs, 33% NPs, 22% PAs) participated in interviews. Factors influencing regimen switch endorsed by ≥50% of providers included resistance, HBV coinfection, eGFR, PWH views, VL, mental health, and weight [Figure 1]; only 22% felt CD4 count was relevant in decisions to switch.

Over 89% of providers reported B/F/TAF or DTG/3TC switch discussions were related to development/anticipation of new symptoms or comorbidities. All providers had patient-initiated switch discussions motivated by individual concerns, advertisements, or social influences [Table 1].

In differential choice analysis, PWH were more likely to be prescribed B/F/TAF if they were viremic, had HBV co-infection, inconsistent clinic attendance, non-adherence, or substance use; providers were more likely to prescribe DTG/3TC when there were concerns about weight or eGFR < 30 [Table 2].

**Figure 1.**
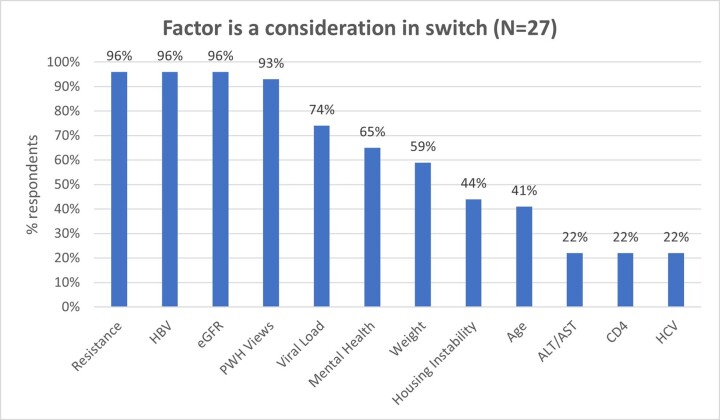
Factors influencing switch to either B/F/TAF or DTG/3TC regimen.

**Table 1.**
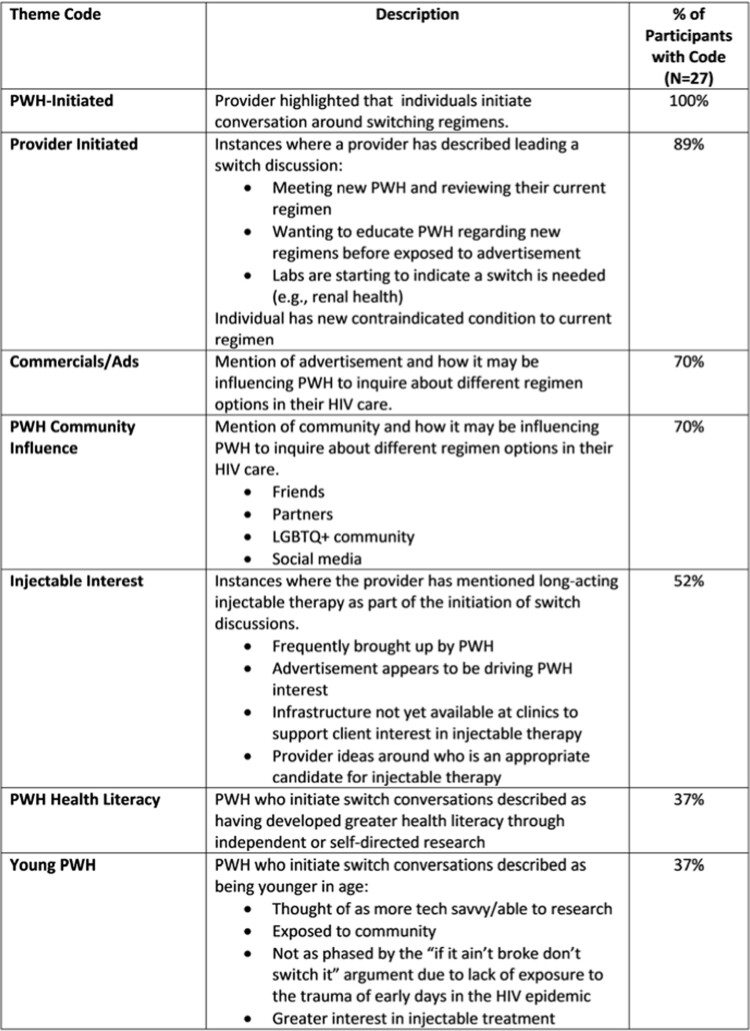
Themes in decisions to consider a switch to B/F/TAF or DTG/3TC.

**Table 2.**
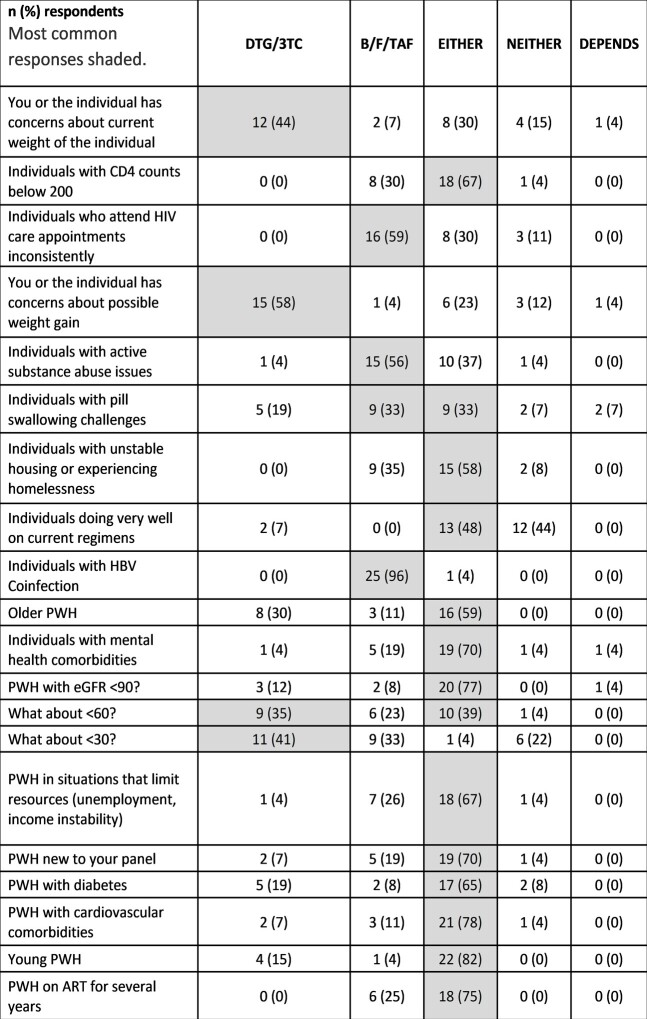
Differential choice based on individual profile.

**Conclusion:**

This mixed methods approach highlights discordance between beliefs and evidence in clinical decision making. CD4 count was not a consideration for ≥75% of providers in interviews, although CD4 < 200 cells/μL was significantly associated with prescribing B/F/TAF in EMR analysis. Provider beliefs surrounding B/F/TAF and DTG/3TC suggested general preferences for B/F/TAF for a broader population, including those with possible adherence challenges, substance use, or resistance, while DTG/3TC was preferred when weight gain or eGFR was a concern.

**Disclosures:**

**Rick A. Elion, MD**, Gilead Sciences: Advisor/Consultant|Gilead Sciences: Grant/Research Support|Proteus: Grant/Research Support|ViiV Healthcare: Advisor/Consultant|ViiV Healthcare: Grant/Research Support **Megan Dunbar, PhD**, Gilead: Employment **Joshua Gruber, PhD**, Gilead Sciences, Inc: Employee|Gilead Sciences, Inc: Stocks/Bonds **Grace A. McComsey, MD**, Gilead Sciences: Advisor/Consultant|Gilead Sciences: Grant/Research Support|Janssen: Advisor/Consultant|Merck: Advisor/Consultant|Merck: Grant/Research Support|ViiV Healthcare: Advisor/Consultant|ViiV Healthcare: Grant/Research Support **Peter Shalit, MD, PhD**, Gilead Sciences: Advisor/Consultant|Gilead Sciences: Grant/Research Support **Janna Radtchenko, MBA**, Trio Health: Employee

